# Creation of a new clinical framework – why women choose mastectomy versus breast conserving therapy

**DOI:** 10.1186/s12874-018-0533-7

**Published:** 2018-07-09

**Authors:** Jeffrey Gu, Gary Groot

**Affiliations:** 1 Department of Community Health and Epidemiology, University of Saskatchewan, Box 7, Health Science Building, 107 Wiggins Rd, Saskatoon, SK S7N 5E5 Canada; 2 Community Health and Epidemiology, College of Medicine, University of Saskatchewan, Saskatoon, Canada

**Keywords:** Framework, Breast cancer, Decision-making, Mastectomy, Breast conserving therapy

## Abstract

**Background:**

Clinical medicine has lagged behind other fields in understanding and utilizing frameworks to guide research. In this article, we introduce a new framework to examine why women choose mastectomy versus breast conserving therapy in early stage breast cancer, and highlight the importance of utilizing a conceptual framework to guide clinical research.

**Methods:**

The framework we present was developed through integrating previous literature, frameworks, theories, models, and the author’s past research.

**Results:**

We present a conceptual framework that illustrates the central domains that influence women’s choice between mastectomy versus breast conserving therapy. These have been organized into three broad constructs: clinicopathological factors, physician factors, and individual factors with subgroups of sociodemographic, geographic, and individual belief factors. The aim of this framework is to provide a comprehensive basis to describe, examine, and explain the factors that influence women’s choice of mastectomy versus breast conserving therapy at the individual level.

**Conclusion:**

We have developed a framework with the purpose of helping health care workers and policy makers better understand the multitude of factors that influence a patient’s choice of therapy at an individual level. We hope this framework is useful for future scholars to utilize, challenge, and build upon in their own work on decision-making in the setting of breast cancer. For clinician-researchers who have limited experience with frameworks, this paper will highlight the importance of utilizing a conceptual framework to guide future research and provide an example.

## Background

### Introduction

Frameworks have been incorporated in research amongst many fields such as population health, public health, and education. However, clinical medicine has lagged behind in understanding and utilizing frameworks to guide research. This has led to research that can be incomplete, redundant, and less effective in drawing conclusions. Furthermore, in our current research environment, establishing methods to increase understanding between disciplines is critical. This article will highlight the importance of utilizing a conceptual framework to guide clinical research, how it can improve the research process, and give an example of creating a new framework to examine why women choose mastectomy versus breast conserving therapy (BCT).

### What is a conceptual framework?

Carpiano and Daley define a conceptual framework as a set of variables and the relations among them that are presumed to account for a set of phenomena [1]. This can range in scope from a modest set of variables, to a capturing a complicated phenomenon such as the WHO conceptual framework for action on determinants of health [2]. Although frameworks set the stage for a scientific inquiry, they do not provide direct explanations for exact outcomes. Understanding a framework’s role in research is often done alongside theory and model, each of which declines in scope, but increases in specificity. Briefly, theory can be broadly thought of as the ‘draft’ explanation as to why a phenomenon is observed, such as why women choose BCT while others do not. A model is a tool used to make specific assumptions about a limited set of parameters and variables that can be tested [1].

A framework has multiple proposes, the most important of which is to help understand the phenomenon of interest in a more complete fashion [1]. It should identify all of the important constructs of a problem, and organizes such constructs in a sensible manner that can be explained. More than one conceptual framework may be relevant to a situation, but often a framework is designed to capture a specific lens or view of an inquiry [3]. It is important to understand the lens and scope a framework was created for and how it might be optimally utilized before adopting it for one’s own research purposes. The latter half of this article gives an example of this process.

A well-constructed framework allows the rest of the research process to follow a coherent structure. A conceptual frame can start by defining the scope of the literature review, as well as aid in its organization. It will assist in variable selection within each construct to be measured. It will guide analysis by allowing researchers to structure their inquiry, and interpret their results based on theory and relationships between different constructs. With the results, researchers are better able to fit their conclusions and add knowledge within the larger context of the overarching framework. One of a frameworks most important purpose is highlighting and communicating how the researcher has chosen to define and structure their phenomenon under study. This can be especially important in aligning disciplines within the context of multi-disciplinary research.

One might ask why so much clinical research fails to be grounded in theory or based on a framework? To start, many clinicians are unfamiliar with a conceptual framework, unless they have completed graduate studies. Of those that have come across the term, many will not understand the full scope and purpose of a framework. Furthermore, clinical research in medicine often follows a course that fails to identify and implement such a guiding framework. A clinician may notice something in their practice, or read a report that inspires a clinical question. This is usually followed by a literature search to identify major works that have been published and a review of existing data on the research query. It is during this process there is an opportunity to come across a framework or theory that other researchers have used. Unfortunately, this is rare in clinical journals, as authors will utilize a traditional literature review to set the stage for their research without considering a framework. As there is no precedence for establishing a framework prior to commencing research, the cycle continues.

In an attempt to capture a holistic snapshot of the existing literature, many researchers will look to a review of the literature, or a systematic review. However, even these are often not based on a framework and can be missing important aspects of a research query. For example, in looking at why women choose mastectomy versus breast conserving therapy, the only published literature review evaluating this topic was by Macbride et al. in 2013 [4]. They synthesized the literature and identified a number of potential factors including patient sociodemographic factors, race and ethnicity, geographical factors, role of the surgeon, role of reconstruction, decision-aids, and influence of BRCA mutation gene [4]. However, despite this being a review article, they did not integrate literature covering key components of this decision-making process including individual patient preference factors and clinicopathological factors such as tumor size. This review did not follow a conceptual framework to guide their work and synthesis of the literature, which may have played a role in missing many important factors.

### Breast Cancer in Canada

Breast cancer is the most commonly diagnosed cancer and the second most common cause of cancer related death for women in North America [5]. Landmark trials have established that breast conserving therapy and mastectomy offer equivalent survival rates and can be viewed as equivalent treatments in early stage breast cancer (ESBC) [6–9]. Treatment for ESBC can therefore be viewed as preference sensitive care, where decision-making between treatment options should vary according to patient preferences and values, but may potentially vary for other reasons as well [10]. However, since the seminal National Institute of Health Consensus Conference in 1999 recommended BCT as ‘preferable’ [11], there have been consistent questions and research regarding quality of care as it relates to regional variation for treatment of ESBC [12, 13].

Significant variations in mastectomy rates amongst regions has resulted in a large body of literature exploring the various factors influencing women’s choice of mastectomy versus BCT [14, 15]. For example, within Canada there has been a large interprovincial variation in mastectomy rates, ranging from 26 to 69% between provinces [16]. Unfortunately the cause of these variations are poorly understood and have gone largely unexplained, primarily due to the lack of a framework to appropriately guide the research question [16, 17]. How do researchers integrate political, psychological, biological, and health care system factors into a research project? What about previous literature, individual belief factors, individual life-circumstances, the physician-patient interaction, and psychological factors? It is in exploring answers to these questions and understanding Canada’s regional variation that has led us to designing a framework that can holistically underpin this phenomenon.

## Methods

### Creating a new framework – Why women choose mastectomy versus BCT

Understanding the factors that influence a woman’s choice between mastectomy versus BCT for ESBC is complicated and multifaceted. At present, there is a lack of a guiding framework within this area. Most of the past research on this topic has been clinically based and not theory driven. Although there are frameworks and theories informing individual choice behaviors on a micro scale, and shared decision-making between the patient and surgeon on a dyad level, there lacks an appropriate framework of scope that can holistically underpin our research. The aim of this article is to present a framework that we have developed to fill this gap in research. The framework was constructed through integrating previous frameworks, theories, models, literature, and clinical research. In the rest of this paper we will introduce our framework, as well as review the key work referenced in creating this framework, and highlight the important elements that were considered.

## Results

**Fig. 1:**
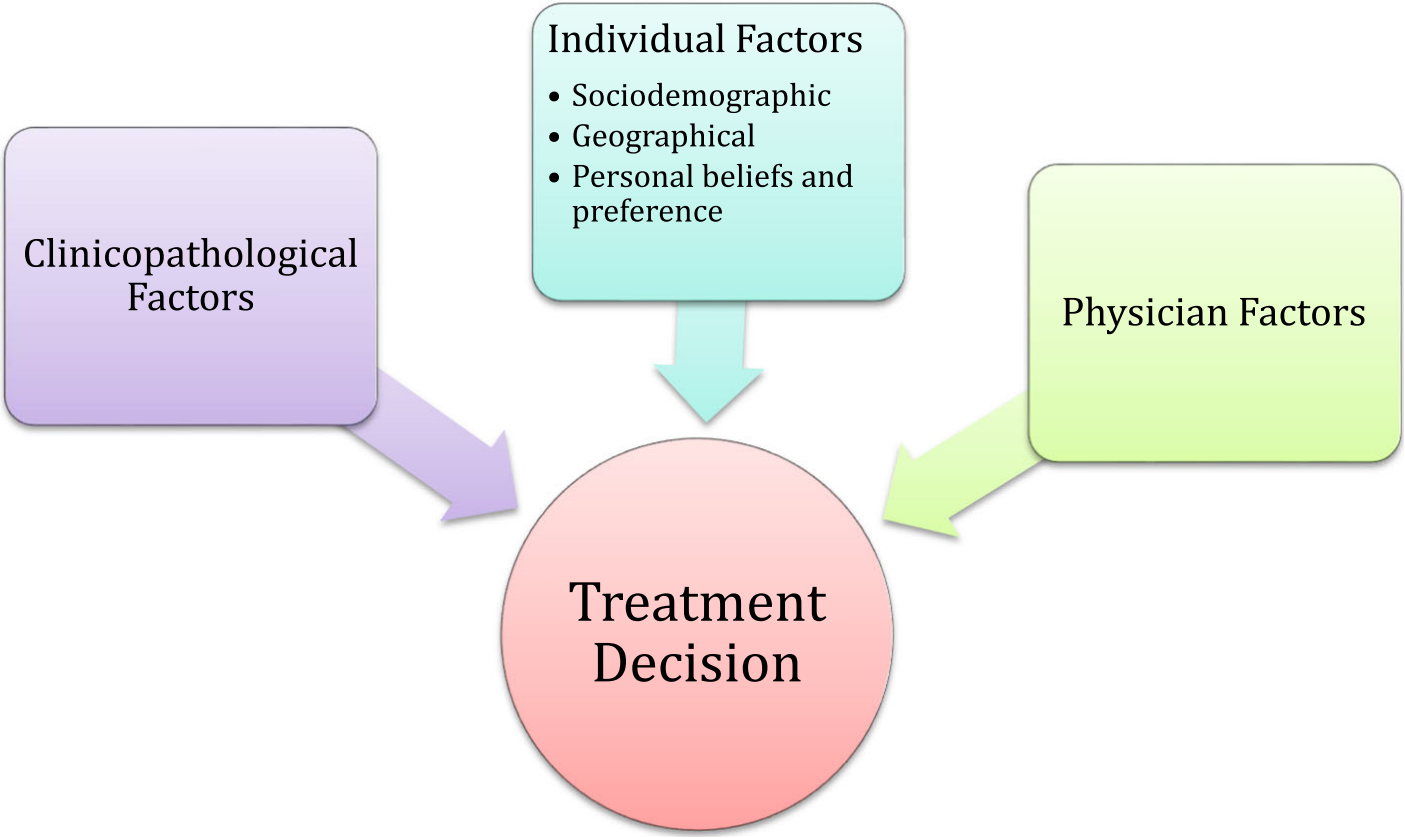
Conceptual framework illustrating the central constructs influencing women’s choice between mastectomy versus BCT

We present a conceptual framework that we have created to illustrate the central domains that influence women’s choice between mastectomy versus BCT (Fig. 1). These have been organized into three broad constructs: clinicopathological factors, physician factors, and individual factors with subgroups of sociodemographic, geographic, and individual belief factors. The purpose of this framework is to provide a comprehensive basis to describe, examine, and explain the factors that influence women’s choice of mastectomy versus BCT at the individual level.

### Clinical literature supporting each construct

#### Clinicopathological factors

Clinicopathological factors are placed in an independent domain because they include tumor biological factors that neither the patient nor the clinician has any direct control over. These factors include tumor size, stage, hormone receptor status, cancer type, and grade. Amongst these factors, larger tumor size, and therefore stage, is the clearest factor that is consistently associated with higher rates of mastectomy [18–21]. More differentiated tumor grades have also been associated with higher BCT rates [19]. These findings are likely multifactorial in reason, with effects on both the individual patient, as well as the surgeon. A larger tumor potentially means a more technically challenging operation, and larger tumors have been associated with increased likelihood of requiring a re-excision [22, 23]. Furthermore, increasing tumor size has been associated with increased local recurrence rates [24–26]. Larger tumor size may also mean a poorer cosmetic outcome. These reasons may potentially bias the physician towards recommending against BCT and decrease the patient’s own belief in the success of BCT.

#### Individual factors

Individual factors can be subdivided into sociodemographic factors, travel related factors, and personal beliefs and preference factors.

Common sociodemographic factors examined in studies include age, socioeconomic status (SES), and race/ethnicity. Age has been examined differently across the literature with discrepant results. The most consistent finding was an increased likelihood of mastectomy in younger age groups, under 40 or under 50 years old [13, 21, 27]. The middle age groups have generally not shown significant findings. The older age groups have shown variable results, with some studies finding increased rates of mastectomy among those older than 70 or 80, while others have shown decreased rates [20, 28–30]. Furthermore, there have been studies in which no association between age and mastectomy rates were found [15, 31]. Multiple studies have found higher SES or other indicators of SES, including education and income, to be associated with increased likelihood of BCT [18, 27, 30, 32]. Ethnicities including African American women, Hispanic women, and Asian/Pacific Islander women have been shown to be independently associated with increased rates of mastectomy [21, 33].

Travel related factors, including distance to a radiation treatment center have shown varying results in terms of their effect on BCT versus mastectomy rates. Although several studies found no difference [32, 34, 35], there are numerous studies that show a decreasing rate of BCT as distance to radiation center increases [13, 32, 36, 37]. In our own qualitative exploration, we hypothesized travel distance would significantly affect rates of BCT due to Saskatchewan’s large rural population. However, our initial qualitative research did not support our hypothesis and further research in Canada would be useful for evaluation [38].

Individual values and preferences may be the most important subset of individual factors, but are the least well studied and hardest to understand. The majority of research into this category has been through simple stand-alone questionnaires, or descriptive qualitative studies. Important personal belief and preference factors influencing choice of mastectomy include fear of recurrence, avoiding radiation, being a more expedient treatment, and avoiding consequences of BCT treatment [15, 29, 35, 39]. Personal belief factors influencing choice of BCT include mastectomy being too radical, surgeon influence, feminine identity, and belief in equivalent survival between BCT and mastectomy [38, 40, 41].

#### Physician factors

Physician related factors have also been examined throughout the literature. Multiple studies have shown surgeon influence and recommendations to be an important factor in treatment decision-making [39, 40, 42, 43]. There have also been various associations identified including sex of physician, case number, individual surgeon practice, subspecialty training, and academic hospital affiliation [4, 37, 44]. Results have differed across studies, with female surgeons being more likely to provide BCT in some studies, but less likely in others [4, 29, 30]. There were a few studies to suggest that individual surgeons have varying practices and can be a predictor of procedural variation compared to their colleagues [43, 45].

### Procedural variation in surgery

Surgical variations between procedures cannot be viewed the same. Wenneberg et al. proposed to group practice variations into 3 categories: effective care, preference sensitive care, and supply sensitive care [10]. Effective care refers to treatments with good evidence behind one intervention, without good alternative options. Examples included colectomy for colon cancer or repair of a hip fracture. Variations in this category generally suggest inappropriate underutilization in lower use areas. Preference sensitive care refers to interventions for problems with more than one acceptable treatment option. Examples include radical prostatectomy, radiotherapy, or active surveillance for prostate cancers and BCT or mastectomy for ESBC. Differences ideally should vary according to patient preferences and values, but could potentially vary for other reasons. Supply sensitive care are services limited by the availability of resources. Examples are availability of physician visits, hospital beds or specialist consultations. Most surgical interventions do not fall under this category. Preference sensitive care represents the largest of the categories for surgery, which includes decision-making between mastectomy and BCT in ESBC.

In 2014, Reames and colleagues published their results of a systematic review focusing on strategies for reducing regional variation in the use of surgery [46]. The review focused on two major strategies to improve consistency and the appropriateness of health care: dissemination of clinical practice guidelines or consensus statements, and shared decision-making tools and decision aids. Results for clinical guideline dissemination demonstrated varied results with some studies showing decreased procedure rates, but others showing no effect or increased rates. Recommendations for procedure choice generally showed a measurable increase in the use of the recommended procedure. With respect to BCT rates, some studies demonstrated a narrowed range of regional variation rates, while others demonstrated a wider range. Decision aids also showed mixed results, with three of five studies not showing a statistically significant change in rate of procedure after administration, and the other two studies demonstrating discrepant effects – one study showed increased rates of BCT while the other showed decreased rates. Although the overall findings show that both clinical guidelines and shared decision-making tools have the potential to reduce the extent of variation in surgical care, these seem dependent on the clinical situation. These findings were confirmed by a recent Cochrane systematic review that also showed decision aids were inconsistent in their ability to change outcome in terms of surgical variation [47].

### Theory of planned behavior

**Fig. 2:**
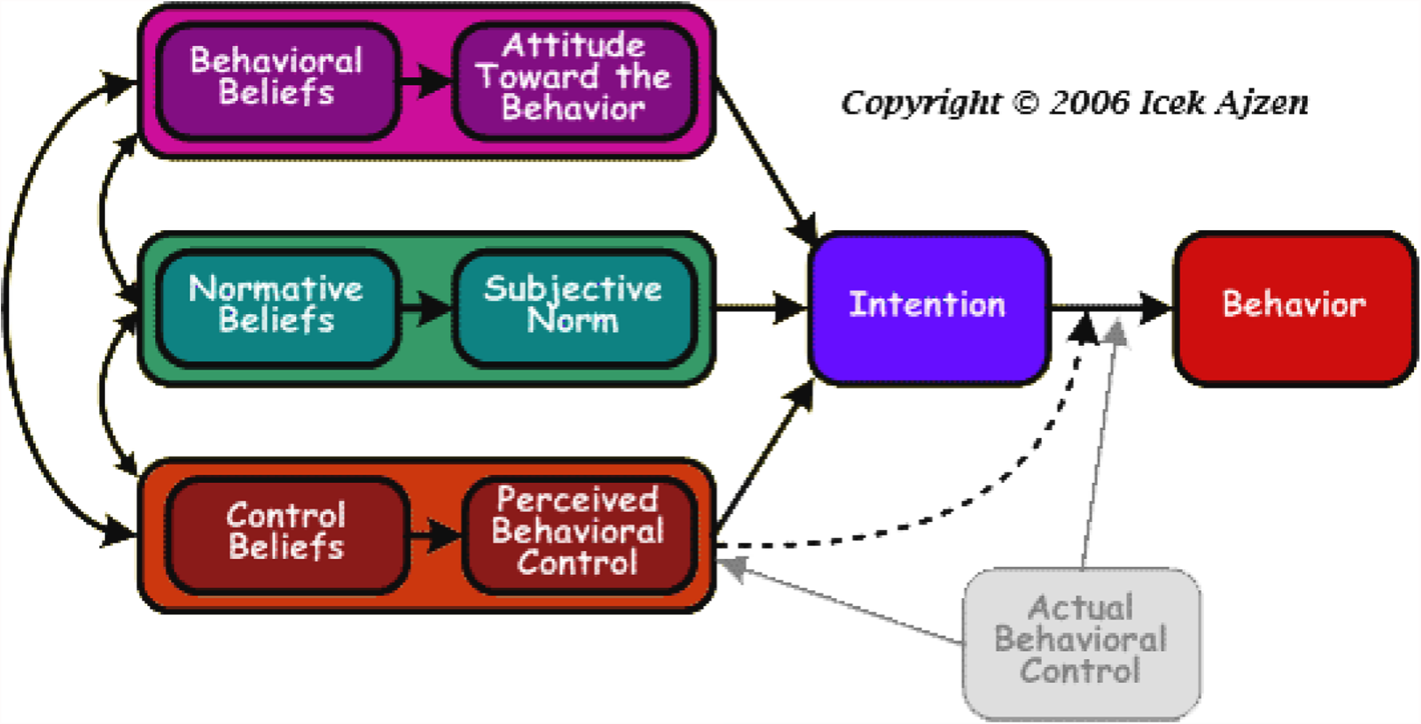
Theory of planned behavior diagram

The Theory of Planned Behavior (TPB) is a conceptual framework that links beliefs and behavior (Fig. 2) [48, 49]. Since its development, it has become one of the most frequently cited and most commonly used theories to predict human functioning and behavior [60]. The theory states that the most proximal determinant of a given behavior is intention, which represents the person’s motivation or decision to act. Intention, in turn, is a function of three sets of belief-based perceptions on behavior: attitude toward the behavior, subjective norms, and perceived behavioral control (PBC). Attitude towards a behavior reflects a person’s overall positive or negative feeling of performing the behavior. This is influenced by behavioral beliefs, which links the behavior of interest to a subjectively expected outcome. Subjective norm reflects the person’s perception of the social pressure to perform a given behavior. This is influenced by normative beliefs, which refer to the perceived behavioral expectations of important referents to the individual such as family, friends, teachers, or their doctors. PBC reflects a person’s overall judgment on whether they have the ability and resources available to engage in the target behavior.

The TPB can be applied to decision-making for women with ESBC. The behavior of choosing mastectomy or BCT can be conceptualized as a planned decision. With respect to applying TPB to decision-making, Ajzen states that: “The TPB emphasis is on the controlled aspects of human information processing and decision-making. Its concern is primarily with behaviors that are goal-directed and steered by conscious self-regulatory processes. From the TPB, expectations that performing a behavior will lead to experiencing pain, pleasure, regret, fear, elation, or other emotions are simply behavioral beliefs, i.e. beliefs about the likely consequences of the behavior, some positive and others negative” [50]. This theory has been utilized to study decision-making in ESBC in past research [43].

**Fig. 3:**
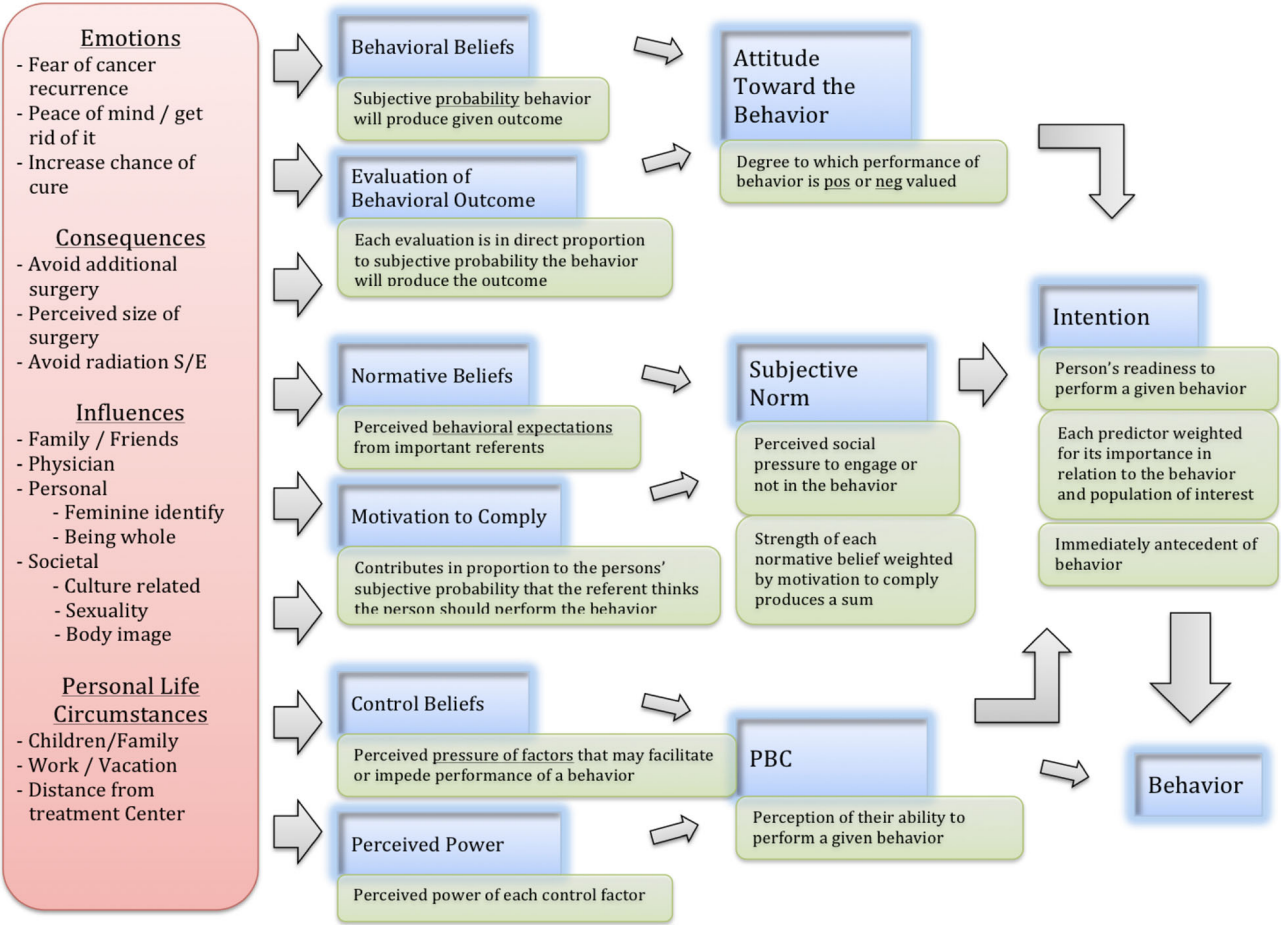
The TPB applied to factors influencing decision-making between mastectomy versus BCT

TPB can be used to conceptualize and theoretically explain how different factors may influence a woman’s choice of mastectomy or BCT. Sociodemographic characteristics can have influences on all three of the TPB belief constructs. Factors such as socioeconomic status or cultural background will directly relate to behavioral and normative beliefs. Personal life circumstances such as work obligations or distance from a treatment center may heavily affect a person’s control beliefs. Influences from close family and friends or surgeon’s recommendations will be among the key referents involved in an individual’s subjective norms. Emotions such as fear of cancer recurrence or peace of mind are often very important determinants of a woman’s choice for mastectomy. These behavioral beliefs can strongly affect the attitude toward the behavior and subsequent behavioral intention. Similarly, an individual’s value of feeling whole or feminine after treatment may have a strong influence on behavioral beliefs. Figure 3 illustrates how the TPB can be used to examine factors influencing the choice between mastectomy versus BCT in ESBC.

The TPB was important in guiding earlier research we conducted. It served as the theoretical foundation in an exploratory qualitative research project titled ‘Understanding Women’s Choice of Mastectomy Versus Breast Conserving Therapy in Early-Stage Breast Cancer’ [38]. The TPB helped frame our qualitative research and organize our analysis, which included creating thematic indices, coding, and creating thematic maps. Although the TPB can potentially account for the full spectrum of factors influencing a patient’s choice of therapy, it is best positioned to closely identify and examine the individual belief and preference factors. As well, the theory was designed to account for all human behavior and is therefore structured at a high level of abstraction, which can make it more difficult for clinicians to relate to in a specific clinical situation such as decision-making in ESBC. We deemed the TPB not specific enough to our research, and felt the need to develop a more targeted and applicable framework. Nevertheless, incorporating aspects of the TPB and our prior qualitative research were critical in creating our new conceptual framework to examine why women choose mastectomy versus BCT.

### Patient-physician shared decision-making

In 1999, Charles et al. presented a framework to examine treatment decision-making that still informs today’s decision-making concepts [51]. There are three models of treatment decision-making included, which are the paternalistic model, shared model, and the informed model. Each of these models are distinguished into three separate steps, or analytic stages: information exchange, deliberation, and deciding on the treatment to implement. The framework describes the general path each of the models follows, and more specifically the behavioral expectations of both physicians and patients for implementing each model. The separate analytic stages of each model make it easy to conceptually distinguish one model of treatment decision-making from another. The framework also recognizes the dynamic nature of decision-making and does not limit one treatment interaction to one model, as the encounter may change as the interaction evolves.

In this framework, decision-making is related to situations where there are several treatment options available with different benefits to risks ratios, and different patient outcomes. Charles identifies four necessary characteristics [52]:

1. At Minimum, both the physicians and patients are involved in the treatment decision-making process.
2. Both the physician and patients share information with each other.
3. Both the physician and the patient take steps to participate in the decision-making process by expressing treatment preferences.
4. A treatment decision is made and both the physician and patient agree on the treatment to implement.

In these appropriate situations, the three models can be divided into three analytically distinct situations to help distinguish their characteristics. Information exchange refers to the type and amount of information exchanged, and the flow of information between physician and patient. Deliberation is the discussion about treatment options, and expression of treatment preferences. The last stage is the actual decision and choice of treatment to implement.

The paternalistic model was traditionally the most prevalent approach to treatment decision-making, generally assuming the physician knows best how to make the best treatment decision for the patient. Information exchange is largely one way in this model from physician to patient. The patient is generally a passive recipient, and information exchange from patient to physician is not seen as important to completing this interaction. During the deliberation stage, the physician will consider the benefits and risks of each option alone or in consultation with other physicians while the patient is passive. The physician makes the final treatment decision alone. Overall this model is labeled paternalistic because it compares to a parent-child relationship where the authoritative figure (the physician) makes the appropriate decision for the patient.

The shared decision-making (SDM) model evolved with clinical medicine as treatment options increased for diseases; this coupled with more emphasis on discussion about tradeoffs between risks and benefits. Information exchange goes both ways in this model. The physician should provide all of the relevant information to the patient to make a decision and the patient should provide information on issues related to the treatment options including values, preferences, social circumstances, and their knowledge about the illness. By providing this information, deliberation can occur within the boundaries and context of the patient’s specific situation. Deliberation should be interactional in nature, ensuring both members have input and are invested in the treatment decision. Expression of treatment preferences is important to this model. The decision on treatment should be agreed upon between both the physician and patient.

The informed decision-making model differs from the SDM model in that the physician limits his or her role to providing information. Information exchange therefore is largely one way, where the physician is the primary source of information for the patient who weighs all of the treatment options and benefits and risks of each option. Beyond information transfer, the physician does not participate further in the decision-making process, leaving the patient to deliberate and make the final choice on the treatment on his or her own. An important fundamental difference from a shared model is the physician should not have an investment in the decision-making process or in the decision made. This is to avoid influencing the patient towards a direction that reflects the physician’s bias of treatment, which may not reflect the interests of the patient.

**Fig. 4:**
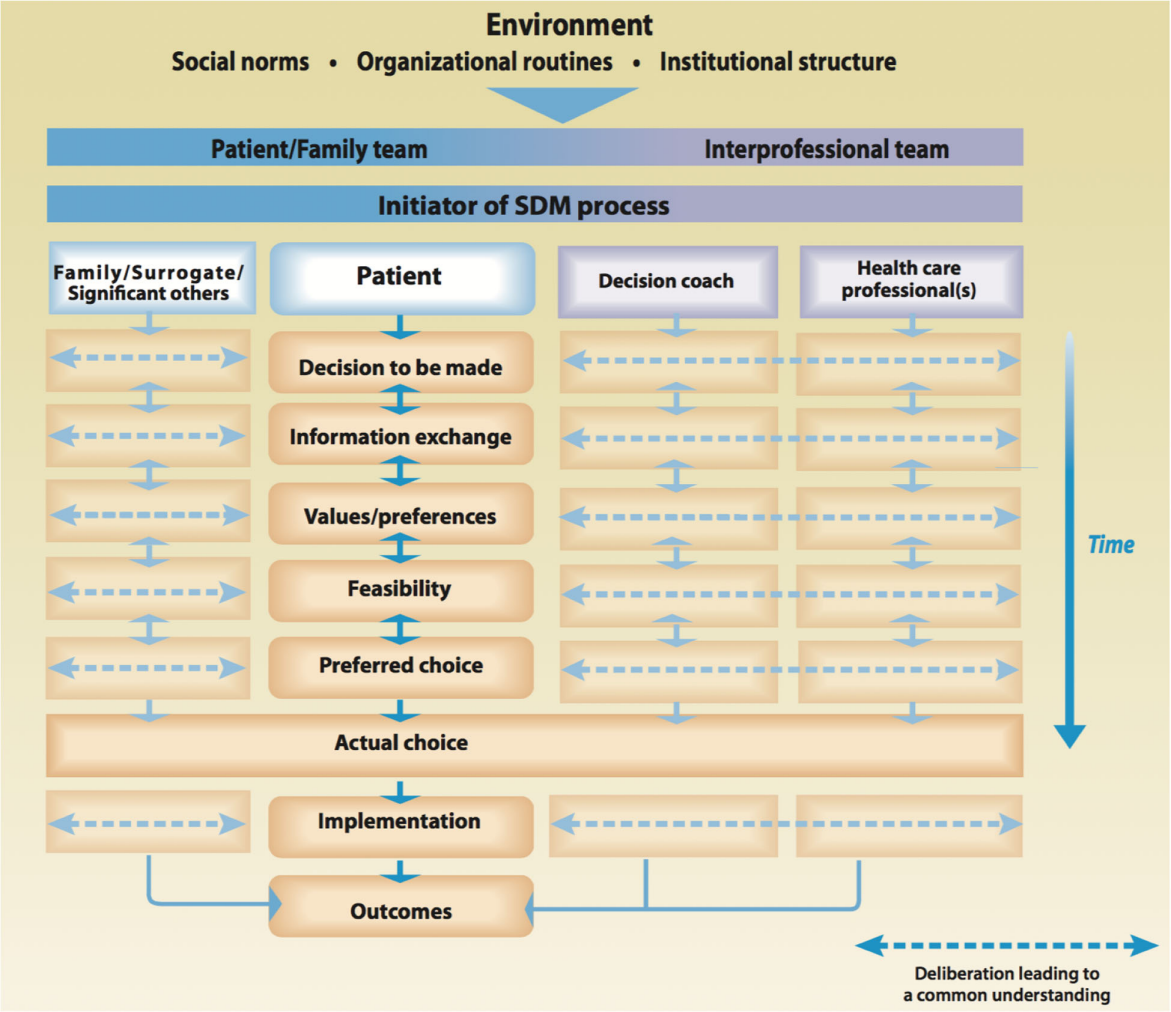
IP-SDM model

There has been a recent push by SDM scholars to create a model that extends beyond the physician patient dyad and promoting an interprofessional approach to SDM. France Legare, Dawn Stacey, et al. proposed a new interprofessional share decision-making (IP-SDM) model to guide patient care [53, 54]. The model is comprised of three levels: the individual (micro) level, the healthcare team/organization (meso) level, and the healthcare system (macro) level (Fig. 4) [65]. For an IP-SDM approach, this model assumes multiple healthcare professionals from different professions collaborate, concurrently or sequentially, to achieve SDM with the patient. The model also assumes the clinical encounters cannot occur independently of the influence of factors from the healthcare system levels. This model has the potential to unify the process of SDM in different healthcare settings and with different health professionals.

The individual level of care is similar to Charles model, except with incorporation of multiple health care professionals, decision coach, and family members’ involvement made explicit through the information, deliberation, and treatment decision stages. The meso level of IP-SDM model is represented by the IP team members and features how this team or organization functions. The macro level represents global healthcare environmental factors: resources, government policies, cultural values, professional organizations, and rules. IP-SDM is a newly proposed model that has had limited development or application into real clinical scenarios and still requires more work to clarify the meso and macro levels [54, 55]. Currently in many North American healthcare systems, the treatment decisions are mostly made with involvement of just one healthcare professional, the surgeon. However, the role and involvement of both medical and radiation oncology in the decision-making process is expanding. There are increased discussions on various lengths of radiation treatments and differing chemotherapy indications, which may increase the role for IP-SDM model in the future.

These models illustrate the complexities behind the patient-physician treatment decision-making. For ESBC, definitive care is centered around surgery and therefore a decision between the patient, with his or her supports, and the surgeon determines treatment choice. The shared decision-making frameworks presented highlight the physician’s influence on the patient’s choice of therapy. Specifically, it brings to light some of the complexities behind the physician-patient interaction that are not generally captured in the clinical literature. The interaction between the physician and patient following the paternalistic model, the shared model, or the informed model will have varying effects of physician influence on the patient, differentiated through the analytic steps. Depending on the style of interaction, there may be different incorporation of patient’s values, beliefs, and preferences into the treatment decision. Apart from the decision-making process as described by the SDM models, the impact of surgeon trust needs to be considered. This can be explained through the normative beliefs construct in the TPB. The influence of trust is especially strong for women who highly value the recommendations and expectations of their surgeon. Recognizing the importance of the patient-physician relationship has led to ‘physician factors’ being a key construct in our conceptual framework. We understand this interactive relationship is complex and differs among individuals, but present here are some of the leading thoughts on how to examine this relationship’s influence on decision-making.

## Discussion

The framework we have presented in this article was designed specific to examining factors influencing women’s choice of mastectomy versus BCT in ESBC only (Stage 1 and 2). It was not designed for patients with ductal carcinoma in situ (DCIS), high risk patient’s such as those with BRCA, or advanced disease such as stage 3 or 4 breast cancer. Although it may be considered for use in these clinical situations, we advise adaptation and critical thinking as the treatment decisions and influencing factors are different compared with ESBC. For example, if this framework were used for locally advanced breast cancer where neoadjuvant chemotherapy is considered, the ‘physician factors’ construct can be modified to ‘multi-disciplinary team’, reflecting a critical change in that decision-making concept [56].

We believe the three constructs created – clinicopathological factors, individual factors, and surgeon factors – are appropriate domains to organize and categorize the potential influencing factors affecting women’s choice. Not only were these constructs developed to be logical for clinicians to visualize and understand as individual domains of factors, but also within the broader context of the framework as a whole. The framework purposefully places the individual in the center, as the treatment is focused on the individual patient. The three constructs have been equally weighted in size because the importance of each can vary depending on the individual. Clinicians should approach each clinical encounter without bias towards one construct.

We recognize that conceptual frameworks are often dynamic entities and invite commentary and alteration as needed.

## Conclusion

We present in this article a conceptual framework that is both unique and novel. It is important to remember that each framework is created to view a phenomenon from a specific lens. These lenses can vary in scope, level of abstraction, and highlight different constructs for the topic at hand. We have created a conceptual framework of why women choose mastectomy versus BCT with the purpose of helping health care workers and policy makers better understand the multitude of factors that influence a patient’s choice of therapy at an individual level. From a research stance, we hope this framework is useful for future scholars to utilize, challenge, and build upon in their own work on decision-making in the setting of breast cancer. For clinician-researchers who have limited experience with frameworks, we hope this paper has highlighted the importance of utilizing a conceptual framework to guide future research and provided a base to begin. For more experienced academics, we invite feedback and hope for continued growth within this field.

## Abbreviations

BCT: Breast conserving therapy
DCIS: Ductal carcinoma in situ
ESBC: Early stage breast cancer
IP-SDM: Interprofessional shared decision-making
PBC: Perceived behavioral control
SDM: Shared decision-making
SES: Socioeconomic status
TPB: Theory of Planned Behavior
